# β-glucans: ex vivo inflammatory and oxidative stress results after pasta intake

**DOI:** 10.1186/s12979-016-0068-x

**Published:** 2016-04-07

**Authors:** Annalisa Barera, Silvio Buscemi, Roberto Monastero, Calogero Caruso, Rosalia Caldarella, Marcello Ciaccio, Sonya Vasto

**Affiliations:** Pathobiology Department and Biomedical Technologies (DIBIMED), University of Palermo, Palermo, Italy; Biomedic Department of Internal and Specialistic Medicine (DIBIMIS), University of Palermo, Palermo, Italy; Department of Experimental Biomedicine and Clinical Neuroscience (BioNeC), University of Palermo, Palermo, Italy; CORELAB, Policlinico Paolo Giaccone, University of Palermo, Palermo, Italy; Department of Biological Chemical and Pharmaceutical Sciences and Technologies (STEBICEF), University of Palermo, Viale delle Scienze, Building 16, Palermo, Italy; Institute of biomedicine and molecular immunology “Alberto Monroy” CNR, Palermo, Italy

**Keywords:** Mediterranean diet, β-glucans, Diet, Inflammation, Oxidative stress

## Abstract

**Background:**

It is well known that Mediterranean Diet can positively influence the health of each individual, in particular it is know that fibers have an important role. However, in Mediterranean cities most people do not have a close adherence to Mediterranean diet. Thus, in our study, we considered fibers like β-glucans that have been added to pasta with a percentage of 6 %. Our study aimed to evaluate the capacity of β-glucans intake on oxidative stress and inflammation in a cohort of middle aged slightly overweight subjects.

**Methods:**

We used a longitudinal study design. The study lasted 30 days during which time, each participant acted with no food restriction. Participants underwent morning fasting blood venous sample for blood chemistry and other biological parameters at the beginning of the study and after 30 days of pasta supplemented with 6 % of β-glucan intake 4 times a week. We performed anthropometric, biochemical, oxidative stress and cytokine analysis at the beginning and the end of study.

**Results:**

After the 30 days of pasta intake we obtained a significant decrease of LDL-cholesterol, IL-6 and AGEs levels.

**Conclusion:**

The results confirmed a capacity of β-glucans intake to lower oxidative stress. Additional longitudinal observation on community-based cohorts are needed to confirm these data and investigate the biological mechanisms through which effects are induced, and to fully explore the therapeutic potential of β-glucans.

## Background

Mediterranean diet (MD) is a set of eating habits around the Mediterranean basin, actually it is not a specific diet but a set of practices of cultivation, fishing, processing and traditions in the preparation and intake of food among the different Mediterranean countries (Spain, Italy, Greece and Morocco). Since 2010, MD is part of the Intangible Cultural Heritage of UNESCO [1]. This set of eating habits consists mainly in a daily intake of whole grains, legumes, fruit and nuts. In addition, there is a moderate intake of fish (along the sea coast), white meat, dairy products and eggs. Intake of red meat and wine is limited compared to the diets of other areas of the world. To ensure the intake of fat, among the people of the Mediterranean basin, intake of olive oil is widespread. Overall, the MD has the following key features: low content of saturated fatty acids, rich in carbohydrates and fibers, high in monounsaturated fatty acids (derived mainly from olive oil), poor animal proteins [2, 3].

It is quite difficult to define the type of fibers in MD because this term expresses a nutritional and physiological concept rather than a class of chemicals. It was originally used to designate the plant residues that are resistant to digestion by enzymes from the intestinal lumen. This definition is still not complete because it does not take into account the heterogeneity of the chemical composition, the diversity of the plant matrix and the physiological characteristics of the multiple components of the fibers. The fibers are distinguished, by an analytical point of view, in soluble and insoluble: soluble fibers act mainly in the first part of the digestive tract (stomach and small intestine), while the insoluble fibers are more active in the terminal part of the digestive tract (large intestine). The fibers also affect the small intestine transit. In particular, soluble fibers delay while insoluble fibers speed up the luminal content. An additional effect of the fibers is their ability to sequester bile acids in the lumen of the ileum: this effect involves, among other things, the absence of the formation of micelles, which are necessary for the absorption of cholesterol and fats. Another possible effect of the fibers is to bind the minerals (Ca, Mg, Fe, Cu, Zn, etc.), reducing their absorption and bioavailability [4].

Despite the beneficial effects of the fibers their intake in the Mediterranean world has sharply declined: a possibility is then to add fibers like β-glucans in the typical foods of the MD such as pasta.

The β-glucans are one of the most abundant forms of polysaccharides found in the cell wall of yeasts, fungi, some bacteria, algae and cereals. All the β-glucans are polysaccharides consisting of linear molecules of D-glucose joined together by glycosidic bonds linear β (1-3) and β (1-4) and differ between them for the length and branched structures. The branches derived from the nuclear chain glycoside are highly variable and the two main groups are branching chains glycosidic β (1-4) and β (1-6). These ramifications appear to be specific, for example, the β-glucans of mushrooms have side branches 1 → 6 while those of bacteria have side branches 1 → 4 [5, 6]. The presence of the bond β (1-3) leads to the formation of folds in the linear chain that allow water to enter; for this reason the β-glucans are classified as soluble fibers. Characteristics of β-glucans are their effect on cholesterol that depends on the ability to form a viscous layer on the surface of the small intestine. The higher viscosity reduces the intestinal absorption of cholesterol and the reabsorption of bile acids. The inhibition of the reabsorption of bile acids can increase the synthesis of bile acids from endogenous cholesterol, and reduces the circulation of cholesterol LDL by about 8 % [7]. A minimum dose of 3 g/day of β-glucans has been suggested reducing the levels of cholesterol in the blood and decrease the risk of cardiovascular diseases [6].

Furthermore, the β-glucans have potent immunomodulatory effects on innate and adaptive immunity. In fact, they have been demonstrated to bind directly to specific receptors of immune cells including Dectin-1, complement receptor 3 (CR3), and TLR-2/6 thus triggering a group of cells of the immune system including macrophages, neutrophils, monocytes, NK cells and dendritic cells [5].

Therefore, the nutraceutical use of β-glucans is an interesting perspective. This action has already been investigated using breakfast drinks [8], biscuits and crackers added with less of 3 % of β-glucans [9]. The aim of the present pilot study was to evaluate the effect of 30 days of regular intake of pasta supplemented with 6 % β-glucans on biological parameters of overweight otherwise healthy individuals.

## Results and discussion

### Hematochemical tests

Physical characteristics and biochemical measurements including cytokines did not change after 30 days of pasta added with β-glucans consumption with the exception of IL-6 blood concentrations as reported in Table 1. The analysis considers weight, BMI, the blood level of glucose, total cholesterol, HDL-cholesterol, LDL-cholesterol, triglycerides, uric acid, creatinine, AST, ALT, γ-GT, total proteins, hematocrit and hs-CRP. In agreement with data reported by a recent meta-analysis [10] the treatment induced a reduction (although not significant) of serum LDL-cholesterol concentrations whereas no significant effect was observed on HDL-cholesterol and triglycerides concentrations or the other parameters considered in this study. In addition, glucose blood concentrations slightly decreased following treatment confirming what reported in previous studies [4]. AST and ALT concentrations were in the range of normality and did not show any significant modification along the study. As stated in the Background section, it is believed that the cholesterol-lowering effect depends on its viscosity in the small intestine, which, in turn, is affected by the molecular weight (MW) and the amount of β-glucans in solution [4]. Body weight and BMI did not vary along the 30 days since the participant were asked to do not modify their nutritional habits.

**Table 1:** Characteristics of the cohort and blood measurements before and 30 days after regular consumption of pasta added with β-glucans

	Before	After	*P* ^*^
Gender (Females/Males)	20/20	20/20	
Age (years)	64 ± 9	64 ± 9	
Body weight (kg)	78.8 ± 3.3	78.7 ± 2.4	0.4
BMI (kg/m2)	28.6 ± 2.1	28.5 ± 1.5	0.2
Fasting blood measurements:			
Glucose (mg/dl)	92 ± 10	91 ± 9	0.3
Cholesterol (mg/dl)	212 ± 32	204 ± 33	0.4
HDL-cholesterol (mg/dl)	56 ± 13	56 ± 11	0.8
LDL-cholesterol (mg/dl)	139 ± 39	127 ± 45	0.03
Triglycerides (mg/dl)	116 ± 69	120 ± 65	0.1
Uric acid (mg/dl)	5.1 ± 1.3	5.5 ± 1.0	0.2
hs-CRP (mg/dl)	0.24 ± 0.20	0.23 ± 0.17	0.2
TNF-alpha (pg/ml)	17.2 ± 13.0	18.3 ± 2.8	0.9
IFN-gamma (pg/ml)	0.6 ± 0.6	1.1 ± 0.8	0.3
IL-8 (pg/ml)	41.4 ± 59.3	45.1 ± 103.8	0.81
IL-10 (pg/ml)	2.3 ± 2.4	1.6 ± 1.7	0.6
IL-6 (pg/ml)	6.3 ± 3.0	7.2 ± 1.7	0.02

All values are presented as means ± SD or in absolute values

^*^Wilcoxon test: *p* > 0.01*p* < 0.5

BMI: body mass index; HDL: high-density lipoproteins; LDL: low density lipoproteins; hs-CRP: high-sensitivity-C-reactive protein; TNF-: tumor necrosis factor; IFN-: Interferon; IL-: Interleukin

### Cytokines

Cytokines are small glycoprotein messengers involved in biological function with several immunological effects. They possess a pleiotropic and potent effector function as in acute and in chronic inflammatory processes and are considered reliable markers of inflammation. The Table 1 shows that, 30 days after the regular intake of the studied food uniquely the IL-6 blood concentrations were significantly decreased. IL-6 is a pleiotropic cytokine capable of regulating proliferation, differentiation and activity in a variety of cell types. In particular, it plays a pivotal role in acute phase responses and in the balancing of the pro and anti-inflammatory pathways. IL-6 is involved in impaired lipid metabolism and in the production of triglycerides. Moreover, it decreases lipoprotein lipase activity and monomeric lipoprotein lipase levels in plasma, which contributes to increased macrophage uptake of lipids [11]. The latter result suggests that a regular intake of the pasta investigated in this study may have an anti-inflammatory effect, probably linked to the immunomodulatory effects of β-glucans [12].

### Oxidative stress analyses

Only few studies described an antioxidant effect of β-glucans [13, 14]. Thus, we have analysed the effects of pasta supplemented with β-glucans intake on oxidized LDL-cholesterol (Ox-LDL), 8-hydroxy-2' –deoxyguanosine (8-OHdG) and 3-nitrotyrosine (3NT) concentrations.

The Ox-LDL represents a modified form of circulating LDL-cholesterol which, accumulates in macrophage by LDL scavenger receptors and play a key-role in the pathophysiology of atherosclerotic plaques. Among the different types of oxidative DNA damage, the formation of 8-OHdG is a marker of oxidative stress [15–17].

**Fig. 1:**
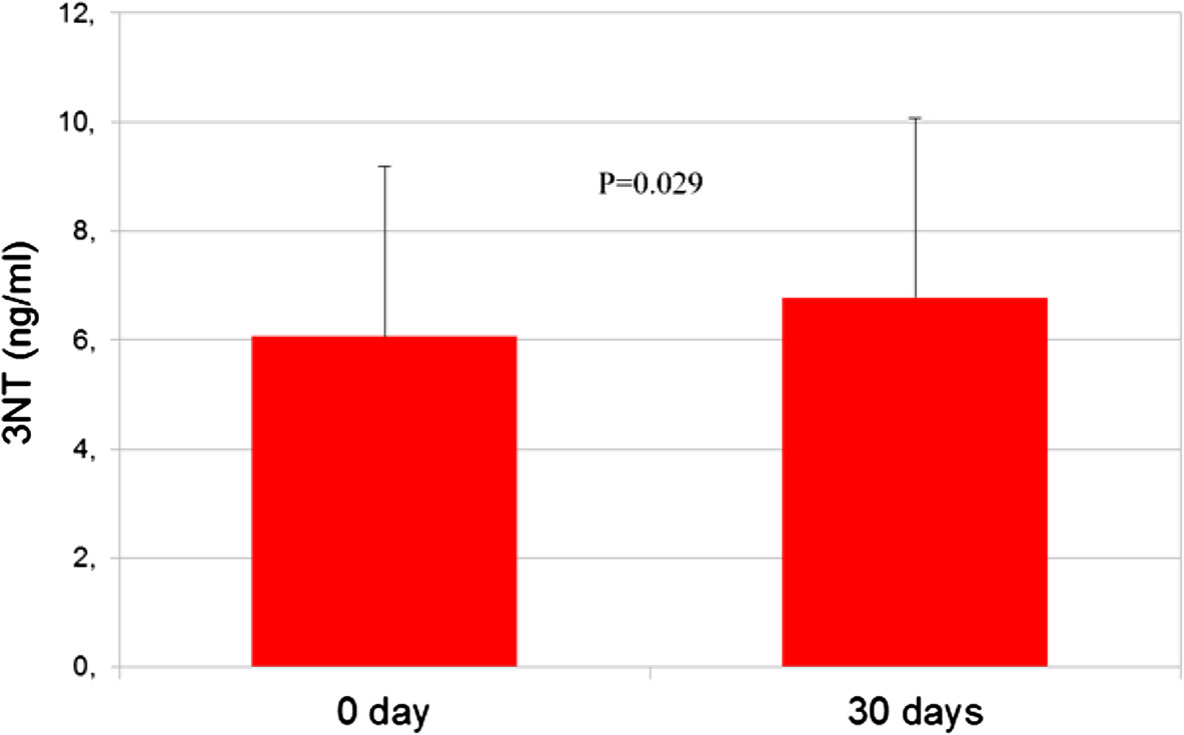
The figure shows 3NT blood levels at time 0 and after 30 days of pasta intake

**Fig. 2:**
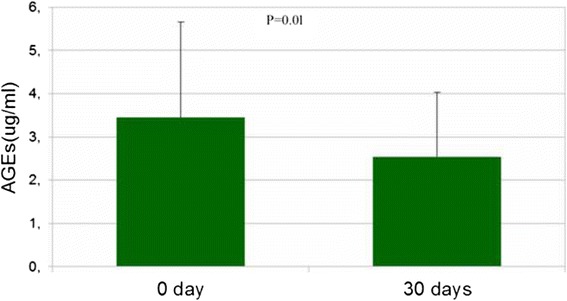
The figure shows AGEs blood levels at time 0 and after 30 days of pasta intake

While all tyrosine residues in proteins might be targets for nitration, the amount and efficacy of tyrosine nitration might vary according to different biological conditions that may vary from the local production and concentration of reactive oxygen species (ROS), antioxidants and scavengers availability to the presence of inflammatory mediators. Fasting blood levels of oxidized LDL were unchanged 30 days after pasta added with β-glucans intake (17.7 ± 16 vs 10.4 ± 6; *P* = 0.9), also unchanged were the fasting blood concentrations of 8OHdG (110.3 ± 133 vs 37.2 ± 32; *P* = 0.07). Furthermore, 30 days after the intake of pasta supplemented with β-glucans blood concentrations of 3NT significantly increased (6.1 ± 3.1 vs 6.8 ± 3.2; *P* = 0.02; Fig. 1) while those of AGEs decreased (3.4 ± 2.5 vs 2.2 ± 1.5; *P* = 0.01. Fig. 2). We cannot exclude that this lack of effect is probably due to the need of longer time of food exposure.

The mechanism that leads to the formation of AGEs starts with a non-enzymatic covalent bond of an aldehyde or ketone group of a reducing sugar with the free amino groups of the proteins and other molecules; subsequently, a series of events consisting in rearrangements and reactions lead to the production of AGEs takes irreversibly place inducing the production of ROS. It has been proposed that the binding and activation of specific receptors, with changing in the extracellular matrix and circulating lipoproteins, which leads to atherosclerosis, realize the vascular toxicity of AGEs.

The present data, although indirectly, are indicative of an antioxidant effect of pasta supplemented with β-glucans.

## Conclusions

It is well known that MD can positively influence the health of each individual, in particular it is know that fibers have an important role. However, in Mediterranean cities most people do not have a close adherence to MD [2]. Thus, in our study, we considered fibers like β-glucans that have been added to pasta with a percentage of 6 %, far higher concentration of commercially available products on the market.

After the 30 days of pasta intake we obtained encouraging results with a significant decrease of LDL-cholesterol, IL-6 and AGEs levels.

According to the results of this pilot trial, we might speculate that a system of well-balanced diet of carbohydrates and fats, as appears to be the MD, may be suitable for helping to correct metabolic abnormalities thus contributing to the clinical management of metabolic syndrome.

Additional longitudinal observation on community-based cohorts are needed to confirm these data and investigate the biological mechanisms through which effects are induced, and to fully explore the therapeutic potential of β-glucans.

## Subjects and methods

### Study design and participants

**Fig. 3:**
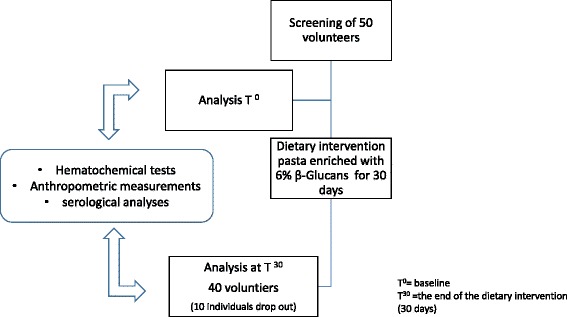
The figure shows the flow chart of the study design

We used a longitudinal study design. The study lasted 30 days during which time, each participant acted with no food restriction (see flowchart, Fig. 3). Inclusion criteria were: range of age 40–60 years, overweight (body mass index [BMI] 25–29.9 kg/m^2^), normal glucose tolerance (fasting plasma glucose < 100 mg/dL), slight dyslipidemia (total cholesterol ≤ 240 mg/dL, HDL-cholesterol 40–59 mg/dL, LDL 130–160 mg/dL, triglyceride level ≤ 170 mg/dL) any treatment for specific disease, including psychotropic drugs and drugs to treat metabolic disorders. Exclusion criteria were: a diagnosis of a severe systemic disorder (including heart disease and hypertension, obesity, diabetes mellitus, dyslipidemia, rheumatological disease, liver, kidney and gastroenterological disorders); psychosis; a history of significant head injury or substance abuse; severe neurological diseases (including stroke, dementia, Parkinson’s disease and other neurodegenerative disorders),eating behaviour disease based on the VAS Questionnaire [18] or under any restrictive dietary treatment. Enrolled subjects underwent a complete internal medicine and neurological examination with trained physicians (SB and RM).

After a complete description of the study, written informed consent was obtained from all participants. Before entering the study, participants were asked not to vary their food and\or physical activity during the period of the study. Accordingly, 40 subjects ended the pilot study (80 % of trial completion) while ten participants abandoned the study before completion for personal reasons.

Participants underwent morning fasting blood venous sample for blood chemistry and other biological parameters at the beginning of the study and after 30 days of pasta supplemented with 6 % of β-glucan intake 4 times a week. Body weight, height and blood pressure of all participants were measured and blood samples were collected at the beginning of the study and after 30 ± 2 days.

### β-glucans preparation

β-glucans extraction, characterization and pasta have been prepared according to Montalbano et al. 2016 [19].

### Blood analyses

Plasma and serum samples were used for hematocrit and chemistry analysis (total cholesterol, LDL-cholesterol, HDL-cholesterol, alanine aminotransferase (ALT), aspartate aminotransferase (AST), glucose, triglycerides, creatinine, gamma-glutamyl transferase (GGT), total protein, uric acid, high sensitivity c-reactive protein (hs-CRP).

### Elisa test

Human Oxidized LDL (MDA-LDL Quantitation) (Oxiselect, cod. STA-369, Cell Biolabs, Inc), Human 3-nitrotyrosine (3-NT) ELISA kit (cod. CSB-E14324H, Cusabio Biotech Co), Human 8-OHdG ELISA Kit (cod. CSB-E10140H, Cusabio Biotech Co), Human advanced glycation end-products (AGE) kit (cod. CSB-E14324H, Cusabio Biotech Co) were used according to manual or data sheet.

### Cytokines assay

Citokines analysis of High Sensitivity IL-6; High Sensitivity TNF-alfa; High Sensitivity INT-gamma; High Sensitivity –IL-8; High Sensitivity IL-10, were measured by -LHSCM000 Human Mag Luminex Performance Assay Base Kit.

### Statistical analysis

The results were analysed by the Biostat 2009 software. Descriptive statistic and non-parametric statistic were used (Wilcoxon test).

## Abbreviations

MD: Mediterranean Diet
BMI: body mass index
MW: molecular weight
HDL: high-density lipoproteins
LDL: low density lipoproteins
hs-CRP: high-sensitivity-C-reactive protein
TNF: tumor necrosis factor
IFN: Interferon
IL: Interleukin
ALT: alanine aminotransferase
AST: aspartate aminotransferase
GGT: gamma-glutamyl transferase
Ox-LDL: LDL-cholesterol
8-OHdG: 8-hydroxy-2' –deoxyguanosine
3NT: 3-nitrotyrosine
AGE: advanced glycation end-products
ROS: reactive oxygen species
